# 

**Published:** 1901-09

**Authors:** 


### BOOKS RECEIVED.

The Diseases of the Respiratory Organs, Acute and Chronic. By William F. Waugh, A. M., M.D., Professor of Practice and Clinical Medicine, Illinois Medical College, etc. Pages 221. Chicago: G. P. Engelhard & Company, 1901. [Price, $1.co, net.]

The Ready Reference Hand-Book of Skin Diseases. By George Thomas Jackson, M.D., Chief of Clinic and Instructor in Dermatology, College of Physicians and Surgeons, New York. New (4th) edition, thoroughly revised. In one 12mo volume of 617 pages, with 82 engravings and 3 colored plates. Philadelphia and New York: Lea Brothers & Co. 1901. Price, cloth, $2.75, net.

Syphilis: Its Diagnosis and Treatment. By William S. Gottheil, M.D., Professor of Dermatology and Syphilology, New York School of Clinical Medicine; Dermatologist to the Lebanon and Beth-Israel Hospitals, the West-Side German Dispensary, etc. Profusely illustrated. Pages 216. Chicago: G. P. Engelhard & Company. 1901. [Price, $1.00, net.]

International Clinics. A Quarterly of Clinical Lectures and Especially Prepared Articles on Medicine, Neurology, Surgery, Therapeutics, Obstetrics, Pediatrics, Pathology, Dermatology, Diseases of the Eye, Ear, Nose and Throat, etc. Edited by Henry W. Cattell, A.M., M.D., of Philadelphia, with the collaboration of John B. Murphy, M.D., of Chicago; Alexander D. Blackader, M.D., of Montreal; H. C. Wood, M.D., of Philadelphia; T. M. Rotch, M.D., of Boston; E. Landolt, M.D , of Paris; Thomas G. Morton, M.D., and Charles H. Reed, M.D., of Philadelphia; J. W. Ballantyne, M.D , of Edinburgh, and John Harold, M.D., of London. Volume II. Eleventh series. Philadelphia: J. B. Lippincott Company. 1901. [Price, cloth, $2.00 each; half-leather, $2.25 each.]

The Diagnostics of Internal Medicine. A Clinical Treatise upon the Recognised Principles of Medical Diagnosis, prepared for the Use of Students and Practitioners of Medicine. By Glentworth Reeve Butler, A.M., M.D., chief of the second medical division, Methodist Episcopal Hospital; Attending Physician to the Brooklyn Hospital, etc. Large octavo, pp. 1077. With five colored plates and 246 illustrations and charts in the text. New York: D. Appleton & Company. 1901.

A Manual of Surgical Treatment. By W. Watson Cheyne, M. B., F.R C.S , F.R.S., Professor of Surgery in King's College, London, Surgeon to King's College Hospital, etc.; and F F. Burghard, M. D and M. S. (Lond.), F. R. C. S., Teacher of Practical Surgery in King's College, London, Surgeon to King's College Hospital, etc. In seven imperial 8vo volumes, with illustrations. Volume V., 482 pages with 145 illustrations. Philadelphia and New York : Lea Brothers & Co. 1901. [Price, cloth, $5 00 net ]

Treves' Surgical Applied Anatomy New edition. Revised by the author with the assistance of Arthur Keith, M. D., F R.C S., in one 12mo volume of 576 pages with 80 engravings Philadelphia and New York : Lea Brothers & Co., Publishers. 1901. [Price, cloth, $2.00 net ]

The Illinois State Board of Health Report of the Sanitary Investigations of the Illinois river and its tributaries. With special reference to the effect of the sewage of Chicago on the Des Plaines and Illinois rivers prior to, and after the opening of the Chicago drainage canal. Secretary, J. A. Egan, M. D.

The Estivo-Autumnal (remittent) Malarial Fevers. By Charles F. Craig, M. D., (Yale), Acting Assistant Surgeon, U. S. Army; Pathologist and Bacteriologist to the U. S. General Hospital, Presido of San Francisco, Cal.; Late Director of the Bacteriological Laboratories of the Sternberg U. S. A. General Hospital, Chickamauga Park, Ga., etc. 8vo., pp. 231. Illustrated by two colored plates and 21 clinical charts. New York : William Wood and Company. 1901.

